# Supplementary figures and images for: A D-2-hydroxyglutarate dehydrogenase mutant reveals a critical role for ketone body metabolism in Caenorhabditis elegans development
Source: PLoS Biol. 2023 Apr 12;21(4):e3002057. doi: 10.1371/journal.pbio.3002057 (PMC10096224; doi:10.1371/journal.pbio.3002057)

A

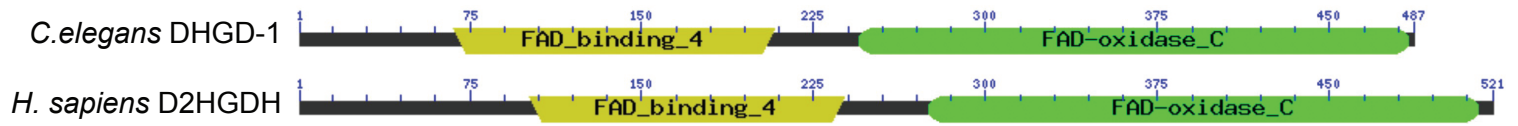

B

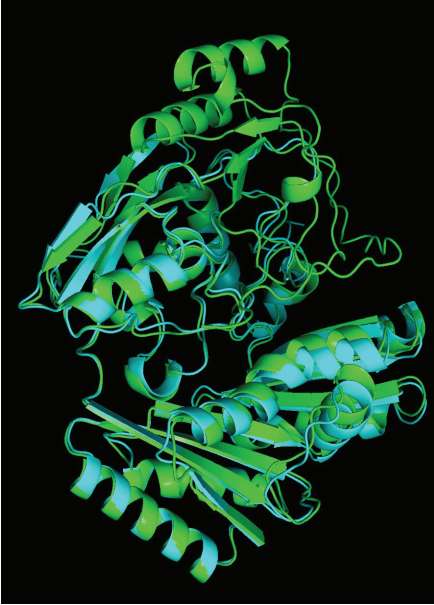

C

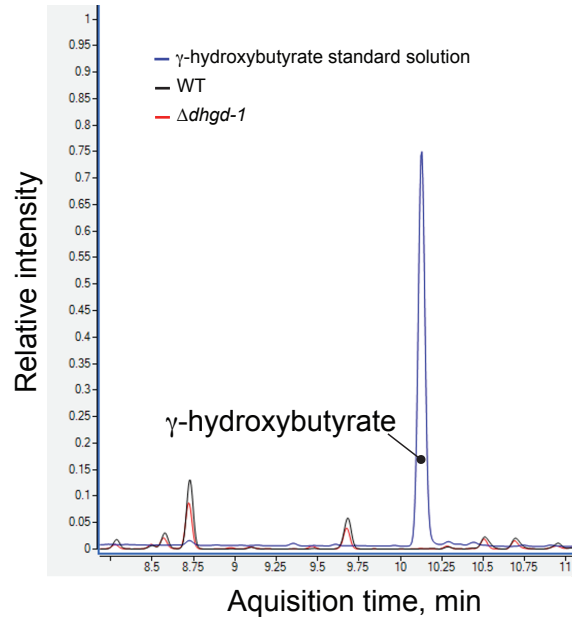

Supplement: S1 Fig — (A) Conserved functional domains in C. elegans DHGD-1 and human D2HGDH according to Pfam database. (B) AlphaFold 3D models of C. elegans DHGD-1 (green) and human D2HGDH (blue) aligned in PyMOL. (C) Chromatograms of WT (black) and Δdhgd-1 (red) C. elegans metabolite extracts compared to 20 mM γ-hydroxybutyrate standard (blue). (PDF) [file pbio.3002057.s001.pdf]

A

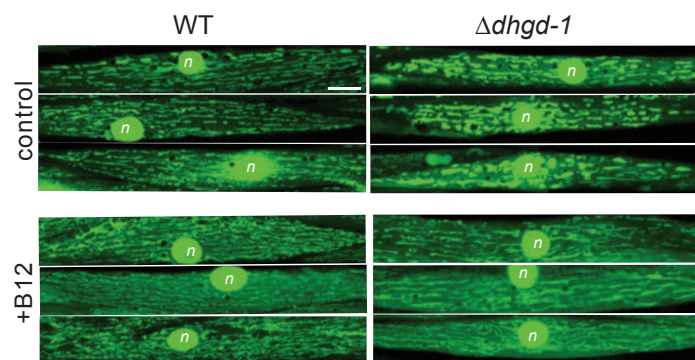

B

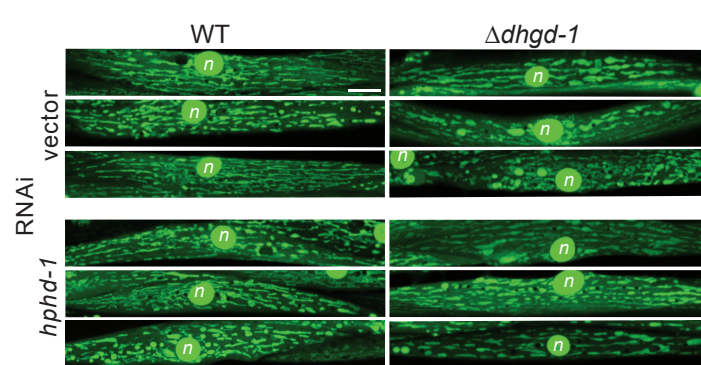

Supplement: S2 Fig — (A, B) Representative images of mitochondria labeled with Pmyo-3::GFPmito in the wall body muscle of L4 larval stage animals. Defects in mitochondrial morphology of Δdhgd-1 mutant C. elegans is rescued by supplementing vitamin B12 (A) but not by hphd-1 RNAi (B). Scale bar 10 μm. Nuclei are marked with “n.” (PDF) [file pbio.3002057.s002.pdf]

Embryos, *E. coli* OP50

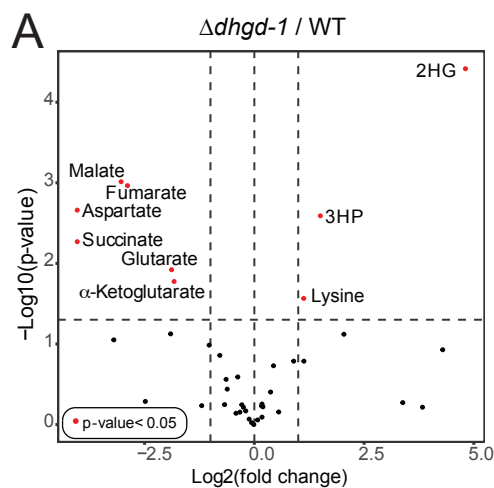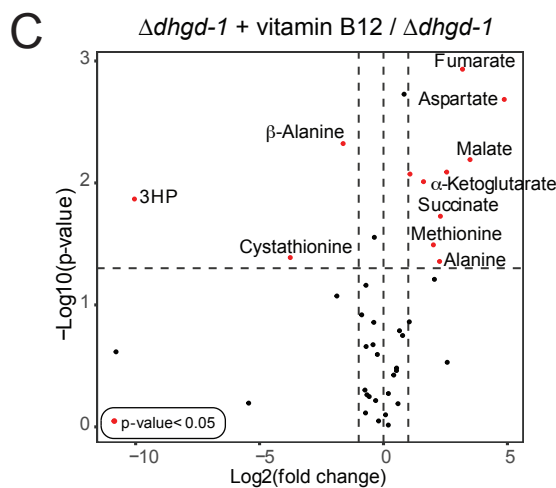

L4, *E. coli* OP50

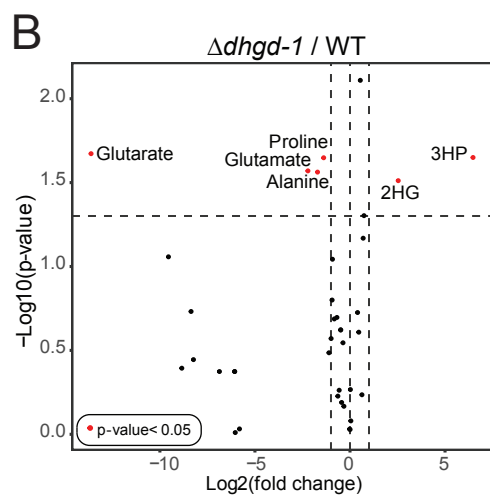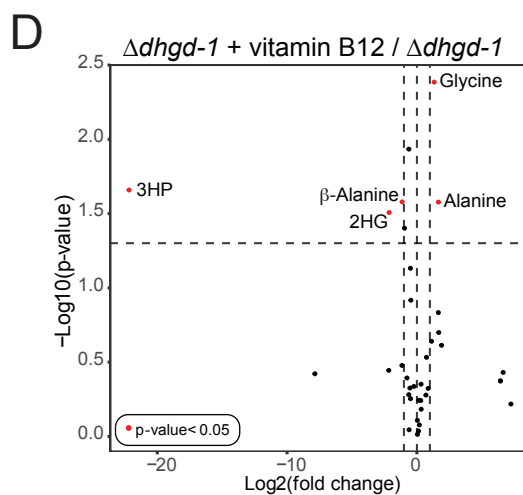

Embryos, *E. coli* OP50(xu363)

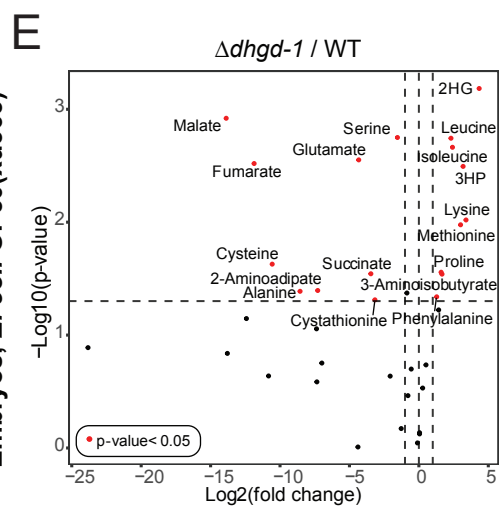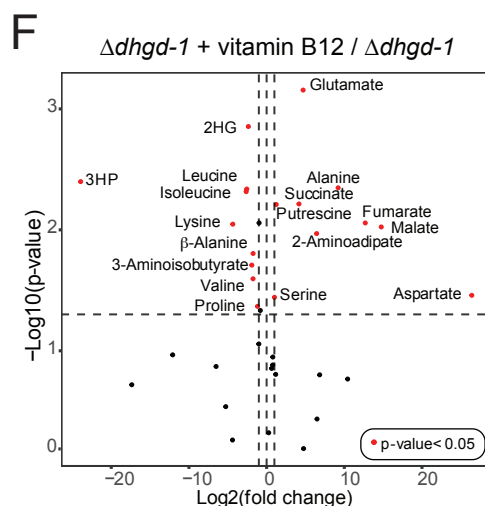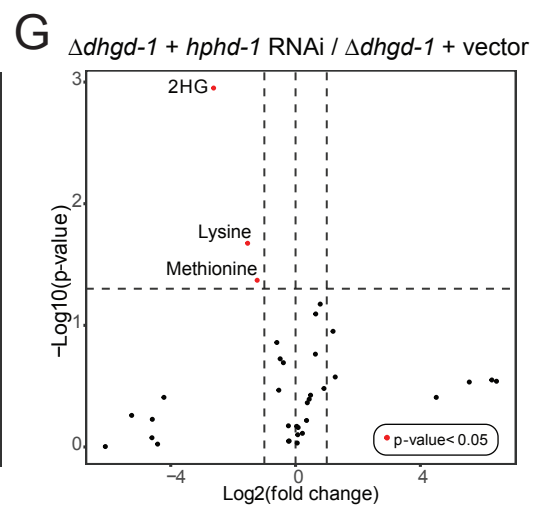

Gravid adults, *E. coli* OP50(xu363)

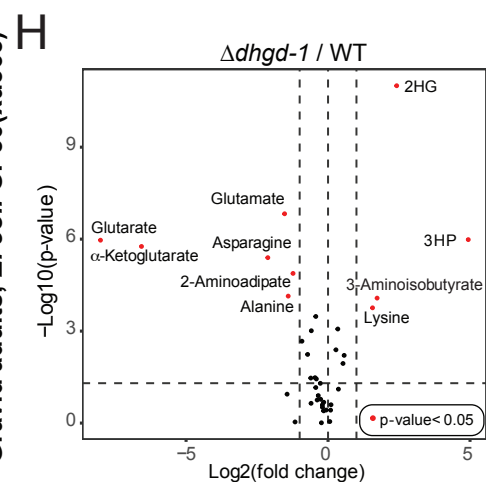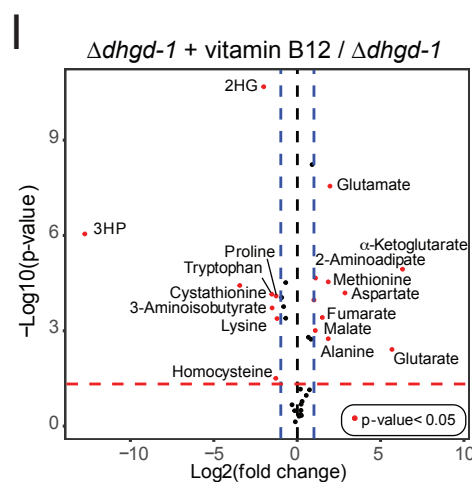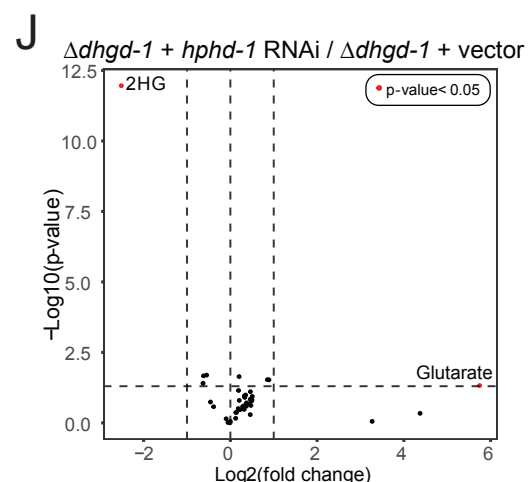

Supplement: S3 Fig — (A, B) GC-MS profiling of metabolic changes in embryos of animals on E. coli OP50 diet: (A) Δdhgd-1 mutants compared to WT C. elegans, (B) Δdhgd-1 mutants supplemented with vitamin B12 compared to Δdhgd-1 mutants. (C, D) GC-MS profiling of metabolic changes in L4 larvae on E. coli OP50 diet: (C) Δdhgd-1 mutants compared to WT C. elegans, (D) Δdhgd-1 mutants supplemented with vitamin B12 compared to Δdhgd-1 mutants. (E–G) GC-MS profiling of metabolic changes in embryos of animals on E. coli OP50 (xu363) diet: (E) Δdhgd-1 mutants compared to WT C. elegans, (F) Δdhgd-1 mutants supplemented with vitamin B12 compared to Δdhgd-1 mutants, (G) Δdhgd-1 mutants treated with hphd-1 RNAi compared to Δdhgd-1 mutants on vector control. (H–J) GC-MS profiling of metabolic changes in gravid adults of animals on E. coli OP50 (xu363) diet: (H) Δdhgd-1 mutants compared to WT C. elegans, (I) Δdhgd-1 mutants supplemented with vitamin B12 compared to Δdhgd-1 mutants, (J) Δdhgd-1 mutants treated with hphd-1 RNAi compared to Δdhgd-1 mutants on vector control. All panels: P-values are Benjamini–Hochberg adjusted. (PDF) [file pbio.3002057.s003.pdf]

A

### 3HP

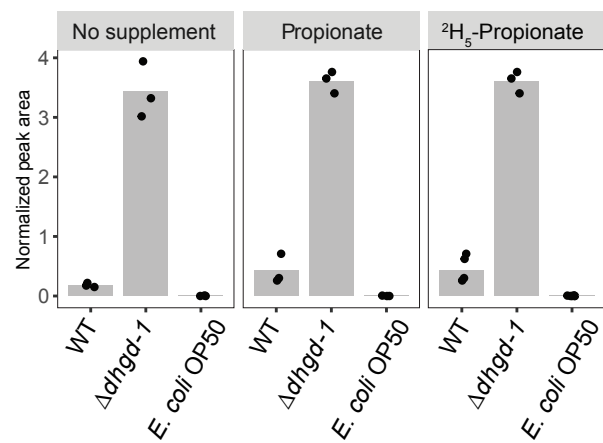

B

### 2HG

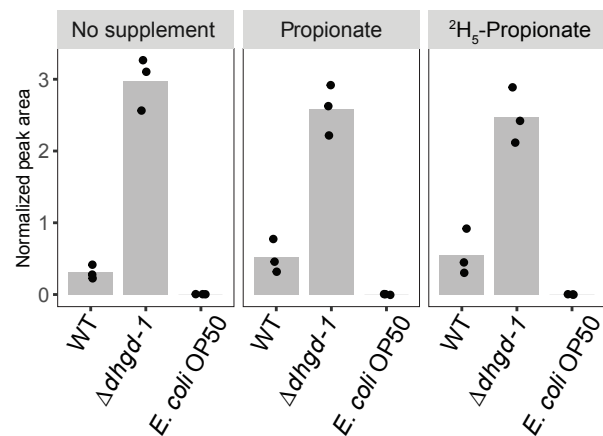

Supplement: S4 Fig — (A, B) GC-MS quantification of 3HP (A) and 2HG (B) in C. elegans and E. coli OP50 supplemented with propionate, 2H5-propionate or untreated. Bars represent mean, each dot represents an independent biological replicate. The data underlying S4 Fig can be found in S1 Data. (PDF) [file pbio.3002057.s004.pdf]

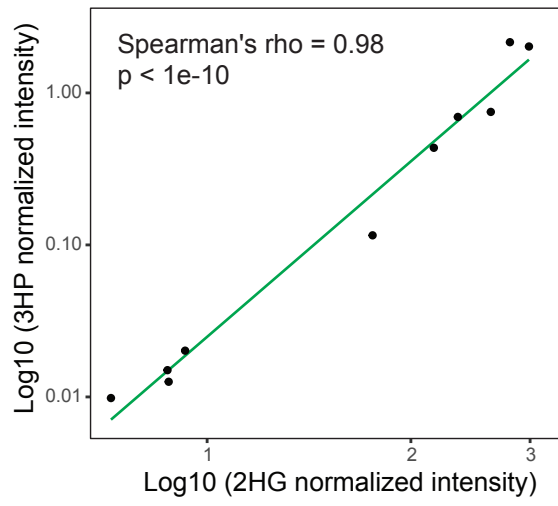

Supplement: S5 Fig — (A) Spearman correlation between GC-MS-quantified 3HP and 2HG levels in Δdhgd-1 animals supplemented with vitamin B12. (B) GC-MS quantification of 2HG in WT and Δdhgd-1 C. elegans fed E. coli OP50 (xu363) diet. The data underlying S5 Fig can be found in S1 Data. (PDF) [file pbio.3002057.s005.pdf]

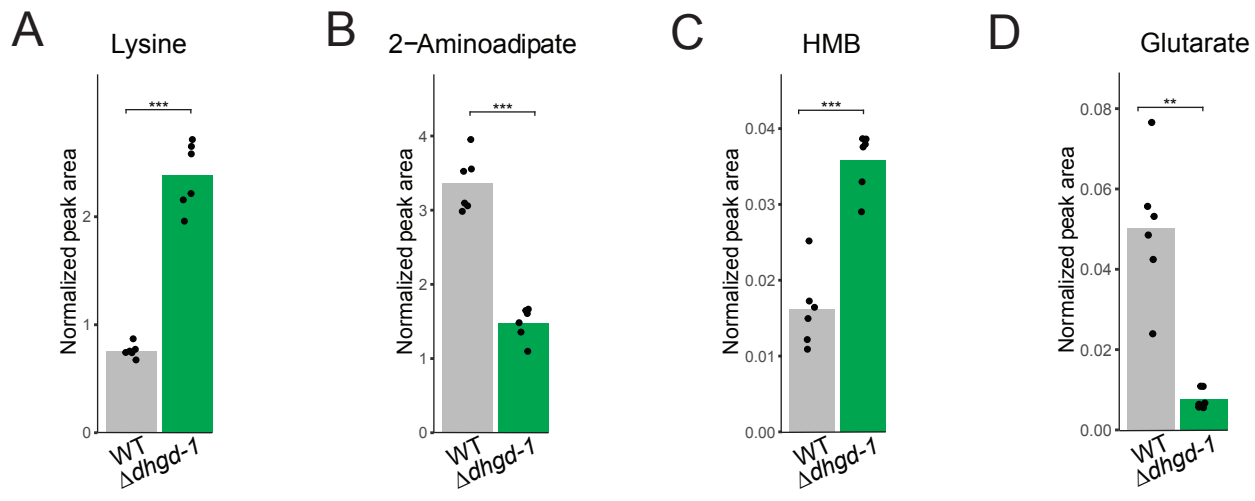

Supplement: S6 Fig — (A–D) GC-MS quantification of lysine (A), 2-aminoadipate (B), HMB (C), and glutarate (D) in Δdhgd-1 mutants and wild-type (WT) animals. Each dot represents an independent biological replicate, **p < 0.01, ***p < 0.001. The data underlying S6A–S6D Fig can be found in S1 Data. (PDF) [file pbio.3002057.s006.pdf]

A

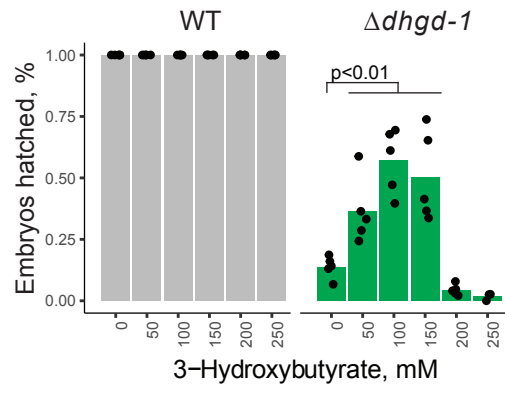

B

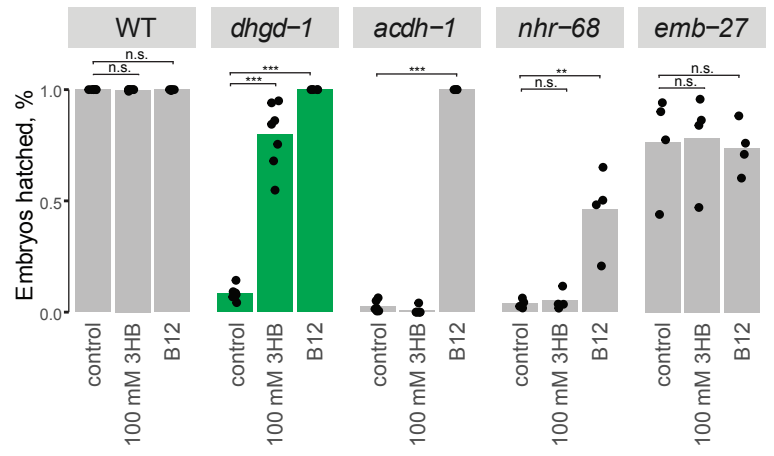

C

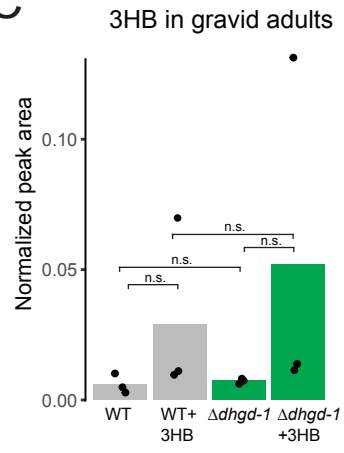

D

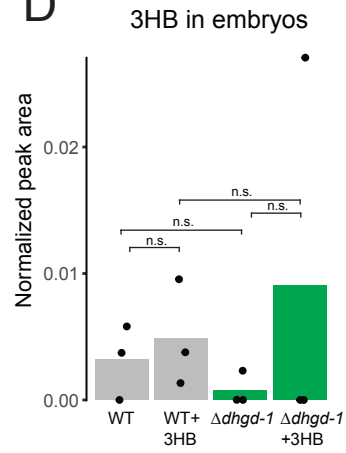

E

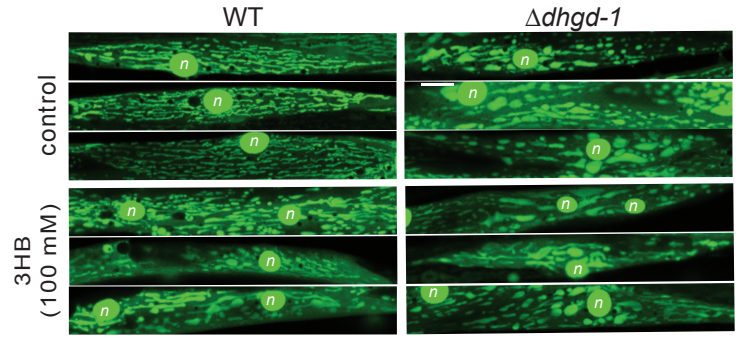

Supplement: S7 Fig — (A) 3HB rescues lethality in Δdhgd-1 mutants. Each dot represents an independent biological replicate and bars indicate means. (B) Effect of 3HB and vitamin B12 on lethality of dhgd-1, acdh-1, nhr-68, and emb-27 mutants. Each dot represents an independent biological replicate and bars indicate means. (C, D) GC-MS quantification of 3HB in WT and Δdhgd-1 C. elegans supplemented with 100 mM 3HB: (C) adults, (D) embryos. (E) Representative images of mitochondria labeled with Pmyo-3::GFPmito in the wall body muscle of L4 larval stage animals. Defects in mitochondrial morphology of Δdhgd-1 mutant C. elegans are not rescued by supplementing 100 mM 3HB. Scale bar 10 μm. Nuclei are marked with “n.” Panels A–D: the means of 3 or more groups were compared with ANOVA, followed by unpaired t test (*p < 0.05, **p < 0.01, ***p < 0.001). The data underlying S7A–S7D Fig can be found in S1 Data. (PDF) [file pbio.3002057.s007.pdf]

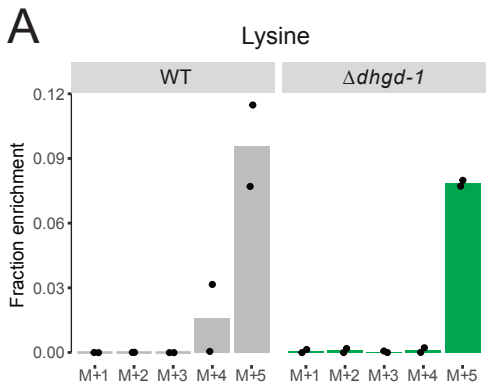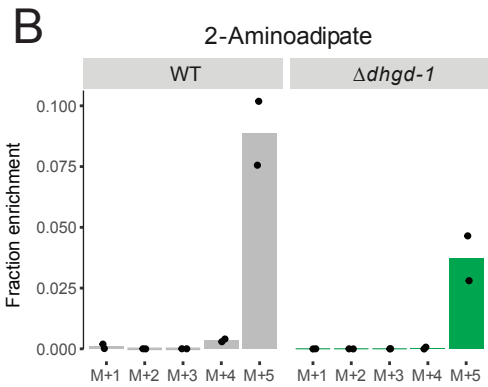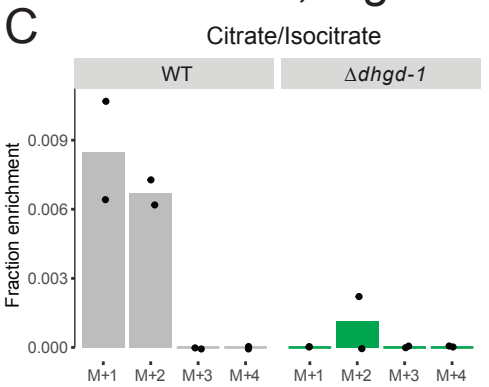

Supplement: S8 Fig — (A–C) Fractional enrichment of lysine (A), 2-aminoadipate (B), and citrate/isocitrate (C) isotopologues in WT and Δdhgd-1 mutant animals fed 13C-labeled lysine. Bars indicate mean of n = 2 biological replicates. The data underlying S8A–S8C Fig can be found in S1 Data. (PDF) [file pbio.3002057.s008.pdf]

## Canonical propionate degradation

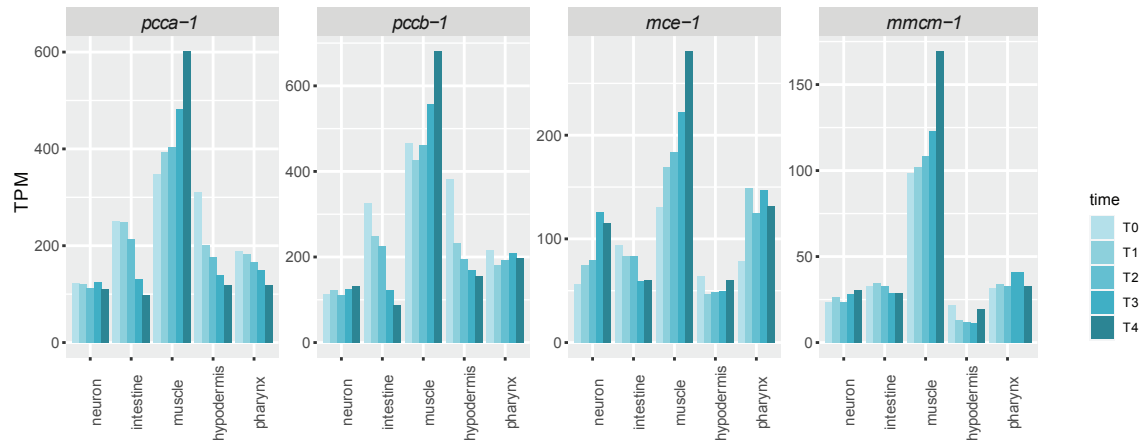

## Propionate shunt

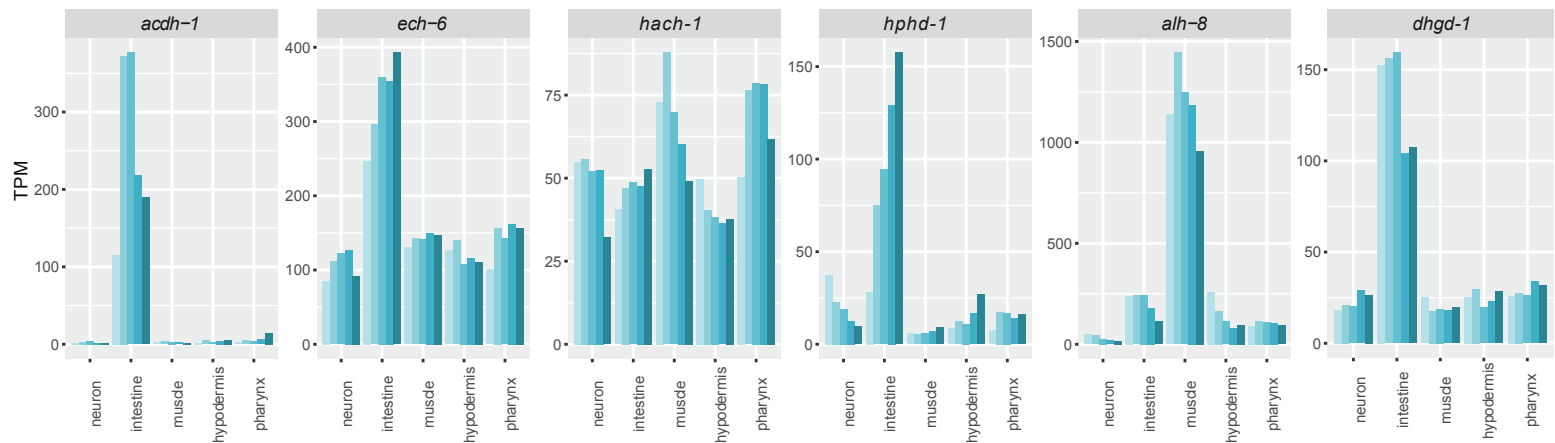

Supplement: S9 Fig — Canonical, vitamin B12-dependent pathway (top) and propionate shunt (bottom) from a published dataset [39]. The data underlying S9 Fig can be found in S1 Data. (PDF) [file pbio.3002057.s009.pdf]
