# Supplementary material for: A D-2-hydroxyglutarate dehydrogenase mutant reveals a critical role for ketone body metabolism in Caenorhabditis elegans development
Source: PLoS Biol. 2023 Apr 12;21(4):e3002057. doi: 10.1371/journal.pbio.3002057 (PMC10096224; doi:10.1371/journal.pbio.3002057)
Supplement: Attachment — Submitted filename: PONOMAROVA REBUTTAL SUBMITTED.docx [file pbio.3002057.s015.docx]

We thank the reviewers and Editor for their helpful comments and suggestions. We were particularly excited by the positive comments, including: “the paper makes a convincing case for the importance of ketone body metabolism in worm development.”

The main point shared by the reviewers is that they requested additional evidence to support our model for ketone body metabolism in *Δdhgd-1* mutant animals.

In the revised manuscript, we provide several additional lines of evidence that support our model:

1. We demonstrate that in addition to the ketone body 3-hydroxybutyrate (3HB) the ketone body acetoacetate (AA) can also rescue embryonic lethality in *Δdhgd-1* mutant animals. This is provided in Figure 4C of the revised manuscript.
2. We performed an isotope tracing experiment with ^13^C-labeled lysine, which is an exclusively ketogenic amino acid. Label incorporation into the lysine degradation intermediate 2-aminoadipate (2AA), a metabolite that is depleted in the *∆dhgd-1* mutant, confirmed that *C. elegans* catabolizes lysine via this pathway (Figure S8). Unfortunately, we could not test if lysine-derived carbon was incorporated into 3HB due to its low abundance and co-elution with 3-hydroxyisobutyrate. We think that 3HB turns over quickly. Indeed, supplementing 100 mM 3HB to the animals did not cause any significant change in its levels (Figure S7C,D).
3. Nevertheless, we were able to detect ^13^C enrichment in citrate/isocitrate, TCA cycle metabolites that are the closest quantifiable intermediate in ketone body oxidation (PMID: 32119410) (Figure S8). Importantly, isotopic labeling of citrate was reduced in *∆dhgd-1* mutants compared to the wild type animals. Together with the accumulation of lysine and depletion of 2AA and TCA cycle intermediates in *∆dhgd-1* animals this result supports our key idea that ketogenic amino acid lysine contributes to energy production and that this is impaired in *∆dhgd-1* animals.

We address all other comments below in blue and hope reviewers and Editor agree our paper is now ready for publication in PLoS Biology.

Reviewer #1: Ponomarova et al. report that a function of F54D5.12 in C.elegans is to suppress levels of D-2-hydroxyglutarate, implying that the product of this gene is a D-2HG dehydrogenase (dhgd). They further report that activity of the gene (which they term dhgd-1) sustains the propionate shunt and enables ketone body synthesis. The paper demonstrates three distinct maneuvers to improve embryonic viability in dhgd-1 mutants: vitamin B12 supplementation (promoting the canonical pathway of propionyl-CoA metabolism), mutation of the propionate shunt enzyme hphd-1, and ketone supplementation. Overall, the paper makes a convincing case for the importance of ketone body metabolism in worm development. A relative limitation of the paper in its current form is that the authors depend heavily on transcriptomics data and FBA rather than direct evidence to infer metabolic activity, particularly with respect to ketone metabolism. Specific comments:

1. The evidence that F54D5.12 is a 2HG dehydrogenase is strong, but with the data presented, it still seems possible that the product of this gene influences D-2HG levels indirectly. Does human D2HGDH rescue viability in dhgd-1 mutants? At a minimum, can the authors show the dhgd-1 protein sequence to demonstrate the presence of domains consistent with oxidoreductase activity? Is there homology with mammalian D2HGDH?

The DHGD-1 protein is 46% identical to human D2HGDH, and the two proteins are reciprocal best blastP hits, which is a commonly used measure of orthology. Both the human and the *C. elegans* protein contain conserved FAD-binding and FAD-oxidase sequences (Figure S1A). Together with our experimental results, notably the buildup of D-2HG in *dhgd-1* mutants, these observations support the conclusion that DHGD-1 is a functional ortholog of human D2HGDH.

We added the following to the revised manuscript: *“DHGD-1 sequence is 46% identical to its one-to-one human ortholog D2HGDH (****Figures S1A*** *and* ***S1B****) and contains same conserved functional domains (****Table S1****)”.*

In addition, we provide the alignment of the human and *C. elegans* protein structures predicted by AlphaFold (PMID: 34265844) as Figure S1B.

2. Can the authors provide more direct evidence of how ketones are produced in the worm? For example, in discussing the results in Fig. 4, the authors suggest that lysine and leucine are major sources of ketone bodies. Isotope labeling experiments using leucine or lysine, and tracking isotope enrichment in acetoacetate or 3-HB, could be used to determine the fraction of ketones produced from these amino acids.

See above.

3. In multiple experiments with three or more groups (Figures 3 and 4), ANOVA should be used, instead of Student's t-test (Krzywinski et al. 2014 Nature Methods).  In the figure legend, only t-test was described.

We thank the reviewer for this suggestion and in the revised manuscript compared the means of three or more groups with ANOVA, followed by the Student’s t-test test to determine which pairs are significantly different from one another.

Minor points:

1. Can the authors comment on whether the gamma-hydroxybutyrate pathway is also active in worms, and if so whether this could be an alternative source of D-2HG? Tracing experiment?

We thank the reviewer for this suggestion. We reasoned that, if gamma-hydroxybutyrate (GHB) is a significant alternative source of D-2HG, then, like 3HP, it should accumulate in *∆dhgd-1* mutant animals. We compared GC-MS measurements of a GHB standard with *C. elegans* samples and did not find any GHB accumulation in wild type or *∆dhgd-1* animals. Therefore, we conclude that GHB is not a significant source of D-2HG in most of the worm body. However, GHB could potentially contribute to 2HG production locally, for example in neurons where GHB may function as a neurotransmitter.

We have added these results as Figure S1C of the revised manuscript and have added the following to the text:“*We asked whether GHB could be an alternative source of D-2HG but could not detect this metabolite in the animal (****Figure S1C****).”*

2. In Figure 1D, why did the L-2HG levels seem to go down in dhgd-1 null animals (compare the green to the blue)?

We apologize for the confusion - Figure 1D shows raw, not normalized data from samples that contained unequal amount of biomaterial. The point is that the **ratio** of D- and L-2HG enantiomers has changed with the accumulation of D-2HG in *dhgd-1* mutant animals.

3. In Figure 1F, the whole error bar seems to float above the grey column. Please double-check.

Figure 1F was corrected to separate error bars corresponding to hatched and a very small non-hatched fraction of the brood size, merging of which caused a “floating” artefact. We have changed the figure in the revised manuscript to avoid this confusion.

4. In the main text describing Figure 2A, the authors stated "…including lower accumulation of glutamate, αKG, succinate…" This would be better stated as "abundance" rather than accumulation, as there seems to be a depletion rather than accumulation of these metabolites.

We thank the reviewer for pointing this out and have revised the text as suggested.

Reviewer #2: This manuscript by Ponomarova et al connects D-2-hydroxyglutarate (D-2HG) production/disposal to propionate and ketone body metabolism in C elegans. Note, this research group has done extensive prior work on the propionate shunt. The D-2HG disposal enzyme, here dubbed dhgd-1, is identified; dhgd-1 mutant worms are generated; D-2HG is elevated in these worms; there is severe embryonic lethality with failure of egg hatching. Metabolomics in dhgd-1 mutant worms demonstrates highly elevated 3-hydroxypropionate (3HP; an intermediate in the propionate shunt) and evidence of impaired leucine/lysine degradation with decrease in downstream metabolites. A model is proposed in which 3HP conversion to MSA by the enzyme hphd-1 is coupled to reduction of alpha-ketoglutarate to D-2HG (and subsequent recycling of D-2HG to aKG by dhgd-1); a very nice labeling experiment with deuterated propionate supports this model. Genetic epistasis experiments show that lethality in dhgd-1
mutant worms can be rescued by deletion of hphd-1; this results in normalization of 2HG levels but 3HP remains high.  Supplementation with vitamin B12 can also rescue lethality in dhgd-1 mutant worms, but this is associated with normalization of 3HP and persistently elevated 2HG. Gene expression analyses indicate that vitamin B12 does not completely suppress propionate shunt enzymes in dhgd-1 mutant worms and that ketone body metabolism enzymes are elevated in dhgd-1 mutant worms regardless of B12 status. Metabolic simulations suggest that dhgd-1 may be important in ketone body metabolism.

Overall, the manuscript is well written and the data are cleanly presented. The genetic experiments and phenotypes are compelling and interesting. The labeling experiment with deuterated propionate is a highlight.  The use of correlation/modeling rather than experiments to connect D-2HG to ketone body metabolism is a major weakness. The following are some suggestions that could be addressed to improve the manuscript at the discretion of the editors.

Major: 

1) The manuscript would be strengthened by metabolomics assessments on dhgd/hphd double KO worms and dhgd KO worms supplemented with vitamin B12. Seeing the effects of these interventions on lysine/leucine degradation products and ketone bodies would help the reader better understand the balance between the propionate canonical/shunt and how it ties in with ketone body metabolism. At present, the connection of D-2HG/d2hgdh with ketone body metabolism is circumstantial. Presumably this would not be overly burdensome to the authors to perform similar analyses as shown in Figure 2A.

We thank the reviewer for this suggestion. We assessed metabolic changes caused by vitamin B12 supplementation and found that it alleviated the perturbation of lysine catabolism (decreased lysine accumulation and increased levels of 2AA and glutarate, Figure S3I ). Similarly, *hphd-1* RNAi increased the abundance of glutarate (Figure S3G).

Because loss of *dhgd-1* causes embryonic lethality, we also analyzed the effect of vitamin B12 supplementation and *hphd-1* RNAi on the embryonic metabolome. In *∆dhgd-1* mutants, *hphd-1* RNAi reduced levels of lysine (Figure S3G), while vitamin B12 reduced levels of lysine and increased 2AA (Figures S3F). Notably, vitamin B12 affected lysine and 2AA only when it also significantly reduced 2HG levels (Figure S3C,F).This underscores the connection between 2HG and lysine catabolism and supports the idea that vitamin B12 does not rescue *∆dhgd-1* mutants through lowering 2HG or restoring lysine catabolism.

Crucially, *∆dhgd-1* mutant embryos have reduced abundance of the TCA cycle metabolites malate, fumarate, and succinate (Figures S3A, E). Levels of all three metabolites were restored by vitamin B12 treatment (Figures S3 B,F), likely due to increased production of succinate via B12-dependent propionate degradation. This result further demonstrates the role of vitamin B12 in rescuing *dhgd-1* mutant energy metabolism.

See above for more evidence that supports our model for ketone body metabolism in *Δdhgd-1* mutant.

2) The discussions about the mechanism(s) of embryonic lethality are difficult to synthesize. Key results are rescue of lethality by hpdh KO with consequent reduction in 2HG levels (Fig 3); discussion here implies lethality related to elevated 2HG. Then vitamin B12 rescues lethality but 2HG remains high. Would help if the authors tried to clearly explain a hypothesis here.  Is it elevated 2HG plus another factor resulting in lethality?

See above. We also revised the manuscript to further clarify our thinking.

3) The failure of 2HG to be suppressed in the dhgd mutants supplemented with vitamin B12 is confusing. How is the 3HP level uncoupled from 2HG here?  If further downstream metabolism of 3HP is somehow enhanced by B12, how does this happen without coupling to D-2HG recycling as the manuscript proposes is necessary?  It would be helpful to perform labelling with deuterated propionate in this setting to see if the D-2HG is indeed coming from the propionate shunt.

We thank the reviewer for this insightful question. Below we describe additional analyses that demonstrate coupling between 2HG and 3HP.

First, since distributions of 2HG and 3HP levels are quite wide *(∆dhgd-1*+B12 samples), we have collected additional samples and calculated rank correlation between 3HP and 2HG. Remarkably, abundance of 2HG and 3HP were strongly coupled (Figure S5A), indicating tight coordination between the two metabolites even in presence of vitamin B12. Increased statistical power from additional samples also revealed a small but significant reduction of 2HG accumulation in the presence of vitamin B12 (Figure 3E). This result shows that vitamin B12 affects levels of both 2HG and 3HP, albeit by different magnitude. Furthermore, in our RNAi experiments that use *E. coli* OP50 strain modified for RNAi compatability (OP50 xu363) vitamin B12 lowers levels of both 2HG and 3HP by more than two folds in adults and embryos (Figure S3F, I).

4) Figure 4 is underwhelming in that mostly circumstantial evidence and modeling is provided, when relatively simple experiments mentioned above would directly confirm connection with ketone body metabolism and support the model proposed in the manuscript.

See above.

Minor:

a) Discuss mechanism of mitochondrial dysfunction as detailed in the Zhou paper.

We have added the following to the Discussion of the revised manuscript: *“3HP impairs mitochondrial health by binding IMMT-1 and inhibiting its membrane-shaping activities” .*

b) For Figure 3, should show mitochondrial morphology of dhgd/hpdh double KO worms or more clearly state whether or not Zhou paper showed this.

We have added a main text reference to the existing supplementary figure S2B which shows that *hphd-1* RNAi does not improve mitochondrial morphology of *∆dhgd-1* animals.

c) Page 9, 2nd paragraph states "This observation confirms that the propionate shunt remains active in the presence of vitamin B12…" Cannot conclude this from gene expression alone. Labelling experiment mentioned above would better support this notion.

We have replaced *”confirms”* with *“suggests”*.

Reviewer #3: This manuscript outlines a novel study centered on the role of the enzyme DHGD-1.  The authors convincingly demonstrate the biochemical and physiological role of DHGD-1 in C. elegans metabolism and development.  The authors then go on to use biochemical and genetic analysis, along with computational measures to elucidate the mechanisms by which DHGD-1 contributes to embryonic viability and mitochondrial function, arguing for a link between DHGD-1 function, the propionate shunt and ketone body metabolism.

While there is some very nice science described in this manuscript, this reviewer does not believe the advances here constitute a broadly interesting and experimentally solid advance that warrants publication in a highly visible broadly interested journal such as PLOS Biology.  This reviewer believes it would make a very nice contribution to a strong, but more specialized journal.

The main reasons for this assessment are that:

1) Even if all of the advances are accurate and well supported, the advances described here are not sufficient to comprise a significant enough and broadly interesting enough advance for publication in PLOS Biology, particularly because it is not clear how much of this study is relevant to other organisms or rather is specific to C. elegans.  This reviewer does not believe it will advance a wider understanding of eukaryotic metabolism relevant to a broad audience.

We respectfully argue that studies on model organisms such as *C. elegans* don’t have to directly translate to other organisms.

2)  While the experiments showing DHGD-1 biological activity and its metabolic contribution to propionate metabolism are convincing and the rescue of dhgd-1 by three different methods is impressive, much of the mechanistic components of the argument are based on computational or database analyses and, while they make sense logically, are still rather speculative in nature.  For example, much of the ketone body argument is built on prior knowledge of metabolic pathways, previously accumulated data, and speculative analyses.  Solid linking of ketone bodies to worm physiology rests on the one experiment showing partial rescue of embryonic lethality by 3HB.  This rescue is not particularly impressive, could mean many different things (outlined below), and really is the only experimental support for the ketone body hypothesis.  Many of the other arguments made in this manuscript about the connection to ketone body metabolism are rather speculative and there are alternative possibilities to account for the experimental observations.

See above.

Significant Experimental Issues:

1. Are the authors analyzing the right material?  Mutation of dhgd-1 causes significant embryonic lethality, yet the authors isolated gravid adults for metabolic analysis.  Therefore, it is not clear whether metabolite analysis primarily reflects dhgd-1 mutant adults or embryos, or both.  This issue is or relevance, for example, the mitochondrial defects were shown in L4 animals, but these L4 animals are worms that have survived the embryo stage and grown up to L4, are the metabolite levels in L4 altered? Or are they only altered in embryos?  These metabolite analyses should really be done with L4 larvae, as well as with gravid adults to resolve these possibilities.

We thank the reviewer for raising this point. To find out if the metabolic changes observed in gravid adults also take place in embryos, we have performed metabolomics of embryonic samples (Figures S3A,E). The results show that *dhgd-1* animals, similarly to gravid adults, have increased levels of 2HG and 3HP. Just like in gravid adults, 3HP levels in embryos were reversed by supplementing vitamin B12 (Figure S3C, F), and *hphd-1* RNAi prevented 2HG accumulation (Figure S3G).

To address the reviewer’s concern about metabolic changes in L4 larvae we have also assessed metabolome of *dhgd-1* animals at this developmental stage. We found that 3HP and 2HG accumulate in L4 *∆dhgd-1,* as in embryos and gravid adults (Figure S3B). This accumulation can also be rescued by vitamin B12 (Figure S3D).

To further clarify the details of L4 mitochondrial morphology experiments we specified in the methods that the L4 animals were of the first P0 generation which was just taken off vitamin B12 and begins to produce inviable embryos.

Overall we found that *dhgd-1* mutation perturbs propionate shunt in L4, gravid adults and embryos alike. These measurements support our thesis that embryonic lethality is caused by 2HG and mitochondrial defects are independent of its levels.

2. The rescue of embryonic lethality by 3HB is not strong enough to make the conclusions made in this paper.  For example, 3HB may simply improve embryonic health via another mechanism.  Because WT embryos hatch at 100%, one cannot assess whether 3HB may simply improve embryonic health.  Perhaps, testing whether or not 3HB generally improves embryonic viability in a range of embryonically impaired mutants would help determine if 3HB nonspecifically or specifically improves embryonic health in dhgd-1 mutants.

We have tested the effect of 3HB supplementation on three additional embryonic lethal mutants; *acdh-1*, *nhr-68* and *emb-27*. While vitamin B12 rescued two of these mutants (as well as *∆dhgd-1*), 3HB only rescued embryonic lethality in *dhgd-1* mutants. This experiment demonstrates that 3HB does not generally improve embryonic health. We have included this data in Figure S7B of the revised manuscript and have added the following to the text: *“3HB rescue is specific to ∆dhgd-1 animals since it does not affect embryonic lethality in other mutants tested”.*

Minor Issues:

1. The authors should describe in more detail where the "co-expression" data are derived from.  In other words, how did the database generate these results.

More details on coexpression analysis were added to the main text. The reference to the recent preprint describing this approach was also added.

2. Figure 3A starts with as schematic, but it is not clear where the schematic is derived from, it is a hypothesis, or an illustration?

We have clarified in the legend caption that Figure 3A illustrates the hypothesis and shows what changes we would expect to see in 2HG and 3HP levels when HPHD-1 and/or DHGD-1 are perturbed.

3. There is no explanation of where the ketone body genes shown in 4B came from, or what they are.

The ketone body metabolism genes were annotated in Walker et al. Genetics 2021 (PMID: 34117752), which we specified in the figure legend.

4. Does 3HB rescue mitochondrial defects?

3HB supplementation does not rescue mitochondrial defects of *∆dhgd-1* mutants. Representative images of three biological replicates are shown in Figure S7E and the following text was added to the revised manuscript: *“3HB supplementation does not change 2HG levels in ∆dhgd-1 mutant animals (****Figures S7C, D****) and does not rescue their mitochondrial defect (****Figure S7E****)”.*
